# P-1335. The in vitro activities of aztreonam-avibactam and cefiderocol against metallo-β-lactamase-producing Enterobacterales isolates collected as a part of the ATLAS Global Surveillance Program, 2021-2022

**DOI:** 10.1093/ofid/ofaf695.1523

**Published:** 2026-01-11

**Authors:** Mark Estabrook, Julie Dickson, Gregory Stone, Katherine Perez, Daniel F Sahm

**Affiliations:** IHMA, Schaumburg, IL; IHMA, Schaumburg, IL; Pfizer, Inc., Groton, Connecticut; Pfizer, Inc., Groton, Connecticut; IHMA, Schaumburg, IL

## Abstract

**Background:**

Aztreonam-avibactam (ATM-AVI) is a β-lactam/β-lactamase inhibitor combination to treat infections caused by Gram-negative organisms, particularly those carrying metallo-β-lactamases (MBLs) and other β-lactamases. Aztreonam is stable to hydrolysis by MBLs and avibactam inhibits Class A, C, and some Class D enzymes. We compared the *in vitro* activities of ATM-AVI and cefiderocol (FDC) against MBL-producing Enterobacterales collected as a part of the ATLAS program (2021-2022).

**Figure:**
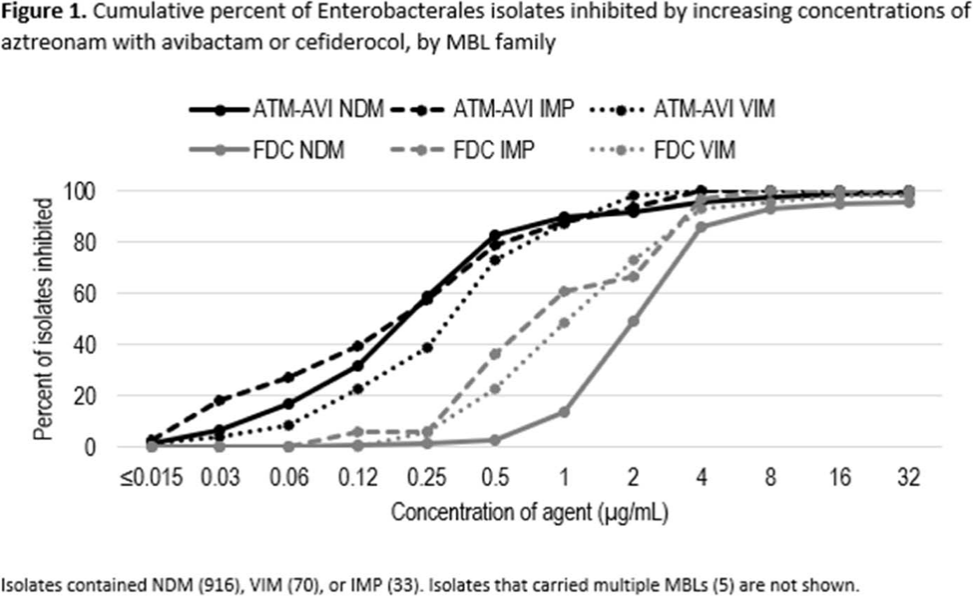

**Figure:**
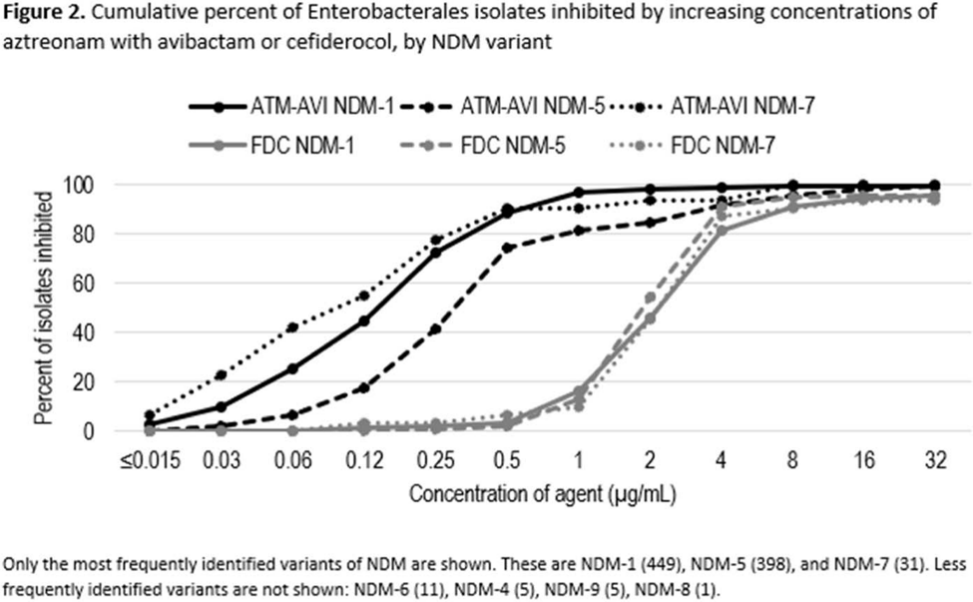

**Methods:**

40,256 isolates from 208 medical centers in 56 countries (excluding Canada and the USA) were collected and tested for susceptibility using the broth microdilution method according to CLSI guidelines. EUCAST 2025 breakpoints were used for ATM-AVI and CLSI 2025 for FDC. Isolates testing with meropenem MIC values >1 µg/mL or *Escherichia coli, Klebsiella pneumoniae, K. oxytoca,* or *Proteus mirabilis* isolates testing with ceftazidime and/or aztreonam MIC values >2 µg/mL were screened for β-lactamase genes by PCR, which were sequenced when identified. WGS was used to characterize isolates collected in China.

**Results:**

1,024 isolates carried an MBL. Isolates carried variants of NDM (916), VIM (70), IMP (33), or a combination of these (5). ATM-AVI was active against more isolates producing MBLs of each family than FDC: NDM, 95.5% ATM-AVI-S, 85.9% FDC-S; VIM, 100% ATM-AVI-S, 92.9% FDC-S; IMP, 100% ATM-AVI-S, 97.0% FDC-S (Figure 1). Isolates that carried NDM as the sole MBL carried NDM-1 (449), NDM-5 (398), NDM-7 (31), or others (22). For isolates carrying NDM ATM-AVI was active against more isolates that carried each variant than FDC: NDM-1, 98.7% ATM-AVI-S, 81.5% FDC-S; NDM-5, 91.7% ATM-AVI-S, 91.0 FDC-S; NDM-7, 93.5% ATM-AVI-S, 87.1% FDC-S.

**Conclusion:**

ATM-AVI demonstrated a higher rate of *in vitro* potency than cefiderocol against isolates that carried MBLs of any family, including all of the most frequently identified variants of NDM.

**Disclosures:**

Katherine Perez, PhD, Pfizer: Stocks/Bonds (Public Company)

